# Mental Health and the Symptoms of PTSD in People with Depression and Anxiety Disorders during the COVID-19 Pandemic

**DOI:** 10.3390/ijerph18115542

**Published:** 2021-05-22

**Authors:** Paulina Beata Golińska, Mariusz Cieślak, Olimpia Hubert, Mariola Bidzan

**Affiliations:** 1 Institute of Psychology, University of Gdansk, 80-557 Gdansk, Poland; mariola.bidzan@ug.edu.pl; 2 Institute of Psychology, University of Lodz, 90-136 Lodz, Poland; mariusz.cieslak@now.uni.lodz.pl; 3 Faculty of Psychology, University of Social Sciences and Humanities, 81-745 Sopot, Poland; olimpia.hubert@gmail.com

**Keywords:** COVID-19, well-being, PTSD, depression, anxiety

## Abstract

The purpose of this study was to examine the severity of post-traumatic stress disorder (PTSD) symptoms related to the COVID-19 pandemic in people with no diagnosis of mental illness, as well as in people who were diagnosed with depression or anxiety. Moreover, this study aimed to investigate the interplay between PTSD symptoms and self-assessed mental health associated with well-being. The 210 participants were divided into 3 groups: mentally healthy, participants with diagnosed depression, and participants with anxiety disorders. To evaluate the subjective well-being of the participants, the Polish adaptation of the Mental Health Continuum–Short Form (MHC–SF) was applied. The Impact Event Scale-Revised (IES-R) was used to measure the severity of PTSD symptoms. At least a moderate worsening of PTSD symptoms was observed in participants of all groups. The results were as follows: healthy participants *M* = 37.35 (*SD* = 18.46); participants with depression *M* = 36.05 (*SD* = 18.02); participants with anxiety *M* = 44.52 (*SD* = 18.08). The participants diagnosed with depression showed the lowest level of mental well-being *M* = 41.58 (*SD* = 15.02). Conclusion: People diagnosed with depression had both the lowest level of well-being and the lowest severity of symptoms specific to PTSD. In all three groups, lower emotional well-being was linked to greater PTSD symptoms.

## 1. Introduction

Definitions of mental health have changed throughout the years. For a long time, mental health was understood as a lack of psychopathology. The current definition approved by the World Health Organization emphasizes well-being as being central to mental health: “Mental health is the state of well-being in which every individual realizes their own potential, can cope with the normal stresses of life, can work productively and fruitfully, and is able to make a contribution to their community” [1].

There are strong relationships between well-being and mental health [2,3]. People who experience psychopathological symptoms significantly more frequently declare lower well-being, which adds up to fewer positive emotions, lower satisfaction with life, poorer functioning, and diminished social life [4,5,6]. However, this does not explain their interrelations and the direction of this dependence. People suffering from mental illness can declare that their mental health is relatively good; thus, the absence of psychopathology is neither required nor sufficient for an individual to positively assess their own life as productive and satisfying [7,8,9].

The two continua model of mental health affirms that positive mental health is related to mental illness, but nonetheless claims that it is a distinct dimension [7]. According to this concept, a person can suffer from a mental disorder (depression, anxiety, etc.) and, at the same time, have a relatively high intensity of positive mental health. This combination of perspectives (the presence of both mental health and mental illness) can lead to better predictions of the psychosocial functioning of a person. In this sense, it can be said that the concepts of mental health and mental illness complement one another [7,10,11]. The current definition of well-being, constructed by Keyes, is consistent with the WHO’s definition and is based on the hedonic and eudemonistic approach, emphasizing positive mental health. According to Keyes, mental health can be understood as emotional, psychological, and social well-being [10].

One long-term study found that an initial high level of well-being, understood as positive mental health in Keyes’ definition, is a protective factor against depression. At the same time, an initial low level of well-being is a predictor of depressive disorders, which can be caused by difficulties in everyday life or experiences classified as traumatic [12,13].

The COVID-19 pandemic, caused by a novel coronavirus first recognized in Wuhan, China, can be considered as such an experience. Soon after the first diagnosed cases, the virus spread rapidly to different parts of the world [14,15]. On 10 March 2020, the World Health Organization classified the spread of the new virus as a pandemic, and several weeks later, the first restrictions on social distancing in Poland were imposed [16]. The stress factors caused by the pandemic can include fear of death, concerns about someone’s own health or the health of their or loved ones, loneliness caused by isolation, concern about the insufficiency of the mental health system, job loss, and the worsening of one’s financial situation. The multitude of stressors caused by the pandemic, especially insufficient information about the spread of the virus and treatment, can provoke negative emotional reactions and are likely to lead to the presence of symptoms characteristic of post-traumatic stress disorder (PTSD) in some people [17,18,19].

Post-traumatic stress disorder can occur following a direct encounter with traumatic stimuli or a life-threatening situation; it is accompanied by severe and chronic stress. The 2009 H1N1 influenza pandemic demonstrated that a pandemic can indeed be a source of PTSD [18,19,20]. Two complementary mechanisms related to PTSD can be distinguished: a biological one (hypersensitivity of the nervous system and the persistence of activated structures in the limbic system) and a psychological one, which has its source in the theory of cognitive schemas [21,22]. Maladaptive schemas can have a negative impact on dealing with difficult situations and can be related to coping strategies. Cognitive schemas are an important element of cognitive stress theory, which assumes that a specific situation can be perceived as stressful based on the primary and secondary emotional assessments of the event [23]. Finding specific circumstances difficult is therefore the result of these mechanisms and the contents of one’s cognitive schemas. In addition, other objective factors are essential to this process, and such factors can be more or less burdensome for different people, such as financial difficulties, potential job loss, or family problems. The subjective assessment of a given situation is a crucial factor in the ultimate perception of the situation as threatening [24]. The aforementioned considerations can contribute to a high level of experienced stress in people who are not being treated for mental illness, as well as in patients with diagnoses of depression and/or anxiety disorders. At the same time, optimism and well-being as a positive assessment of mental health can act as a protective factor against the onset of PTSD symptoms [6,25,26]. According to the authors, the records published so far on the matter of PTSD spread throughout the COVID-19 pandemic apply only to healthcare professionals and the general public. However, there are no available records of research concerning those already suffering from mental disorders such as depression or anxiety disorder, who could be at the greatest risk of mental suffering. Moreover, people without a prior diagnosis of mental illness are characterized by different resistance to psychological distress and can cope with the pandemic situation more or less effectively [7]. 

Hence, the aim of this study was to investigate the severity of PTSD symptoms related to the COVID-19 pandemic in people with no diagnosis of mental illness, as well as in people who were diagnosed with depression or anxiety. Furthermore, this study aimed to examine the interplay between PTSD symptoms and self-assessed mental health related to well-being. Another purpose of this study was to verify if participants with mental illness more often declare languishing and simultaneously will more frequently suffer from PTSD triggered by the pandemic. At the same time, we assumed that regardless of being more prone to PTSD, the participants diagnosed with mental illness declaring high well-being, especially emotional and psychological, will be less likely to suffer from PTSD. Additionally, the two continua model of mental health emphasizes that the two aspects of mental health and mental illness are in fact related, but they represent two different dimensions. Regarding this model, we assume that the participants without mental illness could also be subjected to experiencing PTSD-related symptoms caused by the pandemic, although to a lesser extent than those that suffer from a mental illness. To summarize, the purpose of this study was to indicate the connection between subjective and declarative well-being and the intensification of PTSD symptoms caused by the COVID-19 pandemic.

## 2. Materials and Methods

The study was approved by the Ethics Committee at the Institute of Psychology (University of Gdańsk, no 30/2020). This cross-sectional research was performed during the 18 days when the most restrictive social distancing rules were imposed, 6 April 2020 to 24 April 2020.

After the removal of participants with missing data, a total of 760 individuals participated in this research. Due to social distancing and other restrictions introduced by the government, the research was carried out as an online survey. Google Forms were used for this purpose (a platform that facilitates research preparation and processing). The survey was available on internet forums and social media. Every person had to agree to voluntary participation in the study after getting acquainted with the description of the study. In the socio-demographic survey, two questions were included regarding inclusion criteria: “Were you treated for depression in the last 6 months? (the illness was diagnosed by a psychiatrist)” and “Were you treated for anxiety disorders in the last 6 months? (the illness was diagnosed by a psychiatrist)”. A total of 119 individuals declared to be diagnosed with depression, and 76 participants declared to be diagnosed with anxiety disorders; those that declared to have a diagnosis for both were not considered in further analysis (107 people). Three equal study groups were formed for the purpose of this research: 70 people diagnosed with depression, 70 people diagnosed with anxiety disorders, and 70 people without a diagnosis of mental illness. All the participants were asked an additional question regarding the diagnosis of other mental diseases: “Have you ever been diagnosed by a psychiatrist with depression or anxiety disorders?”, and only those without a history of mental diseases were added to the group of healthy participants. From among the people fulfilling the criteria for any of the study groups, the participants were chosen randomly, for which a random sample formula in SPSS was used.

Information about psychiatric diagnoses is declarative data. In this research, none of the classical psychological mood assessment methods were used, because they are not sufficient for diagnosing depression or anxiety disorders without further psychiatric evaluation. The usage of a structured interview also was not recommended by the ethical committee in the online survey. The mean age was 35.59 years and SD = 10.29 (the range being from 18 to 67 years). All the participants had to meet the age criteria (being 18 or above).

Average age did not differ significantly between the 3 groups, F(2, 209) = 0.599; *p* > 0.5. All data were collected from April 6, 2020 to April 24, 2020, when the most serious restrictions due to the COVID-19 pandemic were in place. 

### 2.1. Methods

A sociodemographic survey consisted of questions regarding sex, age, and education level. Additional questions were asked regarding psychiatric diagnoses and the severity of current symptoms. According to the authors, this is the most objective assessment of the mental state of the tested participants, prior to the pandemic. The additional multiple-choice question regarding concerns was included in subjective consequences of the pandemic (13 items).

To evaluate the subjective well-being of the participants, the Mental Health Continuum–Short Form, Polish adaptation by Karaś, Cieciuch, and Keyes [27] was used. Three subscales contribute to the overall result: emotional well-being, social well-being, and psychological well-being. The higher the obtained result, the higher the level of well-being. In addition, results can be divided into two factors: flourishing (positive functioning and positive emotions) and languishing (low level of positive functioning and fewer positive emotions). In order to be diagnosed as flourishing, a participant must choose the answers “every day” or “almost every day” for at least 6 of the 11 questions measuring psychological and social well-being. In order to be languishing, a participant must answer “never” or “once or twice” at least once to questions concerning emotional well-being and give 6 such answers on the social and psychological well-being scales. The Cronbach alpha coefficient of reliability in this study group was 0.92 for the full scale.

The Polish adaptation of The Impact Event Scale-Revised [28] was used to measure PTSD, a subjective feeling of discomfort as a consequence of a difficult life event. It consists of 22 statements to which the participant responds by choosing one of five possible options on a Likert Scale. The overall result is composed of 3 dimensions: intrusions (8 items), hyperarousal (7 items), and avoidance (7 items).

The average results assigned to the 3 dimensions and the overall PTSD indicator were determined. The sum of points for different dimensions was divided by the number of statements. Analogically, in order to calculate the overall PTSD indicator, the sum of all points on the scale was divided by the total number of statements. The limit value is 1.5 points, relating to individual dimensions as well as the overall indicator of the intensity of PTSD symptoms. The results that exceeded 1.5 points on the scale indicate at least moderate intensification of the symptoms.

The Cronbach alpha coefficient of reliability in this study group was 0.93 for the full scale, and for the individual scales, it was as follows: intrusion 0.90, hyperarousal 0.83, avoidance 0.78. The participants were asked to relate their answers to the COVID-19 pandemic.

### 2.2. Statistical Analysis

Data management and analysis were performed using SPSS, version 26. Descriptive statistics were generated for all the variables included in this research (sociodemographic, perceived consequences of the pandemic, well-being, and PTSD), including the division of the participants into 3 study groups. A one-way analysis of variance for independent samples was performed in order to verify the statistically significant differences of average well-being and PTSD. Additionally, the Pearson correlation coefficient was calculated in order to verify the connection between well-being and PTSD. At the end, a linear regression was carried out, in which all the variables were included.

## 3. Results

### 3.1. Descriptive Statistics and Group Comparisons

A total of 210 Polish people participated in this research. Detailed characteristics of all groups are presented in Table 1 below.

**Figure 1:**
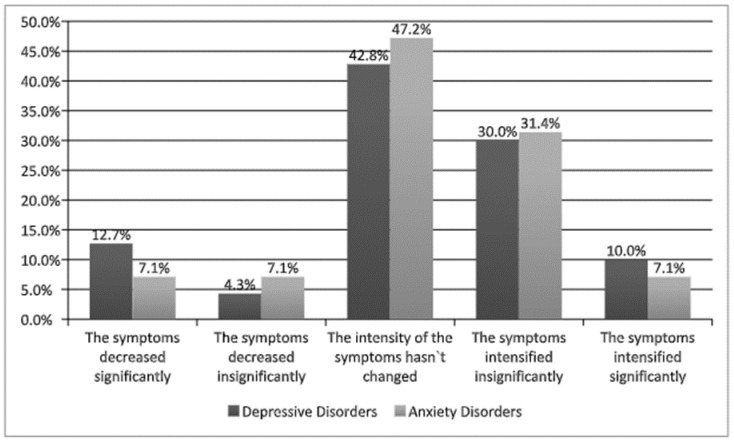
The intensity of symptoms in individuals diagnosed with depression or anxiety disorders during the COVID-19 pandemic.

The participants with diagnoses of depression or anxiety disorders were asked to indicate the current severity of their symptoms. The results can be seen in Figure 1 below.

### 3.2. Intensity of Symptoms and Concerns of Pandemic

The obtained results might suggest that the declaration about the intensification of depression or anxiety symptoms in all tested groups did not increase or decrease significantly; however, almost one-third of the participants experienced a slight intensification of their symptoms due to the COVID-19 pandemic.

The tested participants had different concerns related to the pandemic as well. The frequency of concerns indicated is presented in Table 2. The data is summarized based on the average number of indicated concerns, which was highest in the group with depression.

### 3.3. The Differences Between Study Groups Regarding PTSD and Well-Being

The mean severity of well-being was subsequently examined in all the study groups. A one-way analysis of variance for independent samples was performed to verify the statistically significant differences of average well-being in the study groups.

The carried-out analysis confirmed that the differences between the compared groups were statistically significant. According to these results, the healthy participants, people with depressive disorders, and those with anxiety disorders differed from one another regarding the average level of their well-being and their well-being in three dimensions. The results can be observed in Table 3. 

The carried-out analysis of variance demonstrated the difference between study groups in their general well-being and one of its dimensions (social). The participants with depressive disorders were characterized by a significantly lower overall result and lower social level of well-being in comparison with participants without a diagnosis. Other comparisons in this study were not considered statistically significant.

Subsequently, the participants were divided based on whether they were flourishing or languishing. A total of 31 (11%) participants were flourishing and 51 (18%) were languishing. A breakdown for each subgroup is presented in Table 4.

A one-way analysis of variance for independent samples was performed once again to verify the significance of differences in average PTSD severity. The analysis showed that the compared groups differed significantly, which means that healthy participants, people with depressive disorders, and those with anxiety disorders differed from each other regarding the average level of the severity of their PTSD symptoms and 3 of its components. Table 5 represents the obtained results.

The analysis of variance demonstrated that the compared groups differed from each other significantly in their general severity of PTSD symptoms and its components. The participants with anxiety disorders were characterized by a significantly higher general level of severity of PTSD symptoms in comparison with the participants suffering from depression. Other comparisons in this study were not considered statistically significant.

### 3.4. The Links between PTSD and Well-Being in Study Groups

The next phase of the analysis aimed to identify the connection between well-being and experienced PTSD symptoms in all tested groups. The results obtained are presented in the tables below (Table 6).

The results obtained for individuals with no diagnosis showed a moderate negative correlation between emotional well-being and the overall PTSD result. A weaker negative correlation was also observed between overall well-being results and PTSD. The same applied for intrusion and hyperarousal. The remaining correlations were not statistically significant. The results obtained for participants with depression showed a moderate negative correlation between emotional well-being and the overall PTSD result and hyperarousal. A weaker negative correlation was observed between emotional well-being and intrusion. The overall result of well-being correlated negatively with the overall PTSD result and hyperarousal. The remaining correlations were not statistically significant. The results obtained for participants with anxiety showed a weak negative correlation between the overall PTSD result and overall well-being, as well as two of its components: emotional well-being and social well-being. Emotional well-being correlated negatively with all three PTSD components. There were rather weak negative correlations of both social well-being and overall well-being with intrusion and hyperarousal. The remaining correlations were not statistically significant.

The following phase of the research analysis focused on the relationships between well-being and PTSD. A linear regression analysis was first carried out on the overall well-being results and then on its components in all three tested groups. The results are presented in Table 7, Table 8, Table 9, Table 10, Table 11 and Table 12 shown below.

The linear regression analysis on individuals with no diagnosis indicated the significant role of overall well-being and emotional well-being. It can be concluded that the higher the level of overall and emotional well-being experienced by a participant, the less severe the PTSD symptoms were.

The linear regression analysis for participants with depression indicated the significant role of overall well-being and emotional well-being. It can be concluded that the higher the level of overall and emotional well-being experienced by a participant, the less severe the PTSD symptoms were.

The linear regression on participants with anxiety disorders indicated the significant role of overall well-being and emotional well-being. It can be concluded that the higher the level of overall and emotional well-being experienced by a participant, the less severe the PTSD symptoms were.

## 4. Discussion

The aim of this study was to analyze the relationships between self-assessed well-being and experienced severe stress, characteristic to post-traumatic stress disorder (PTSD) during the 2020 COVID-19 pandemic. Three groups of participants were tested: individuals without mental disorders, individuals diagnosed with depressive disorder, and individuals diagnosed with anxiety disorder. A total of 40% of the participants diagnosed with depression and 30% of those diagnosed with anxiety disorder declared that their symptoms had intensified around the time when the most radical social distancing limitations were implemented during the pandemic.

Depression and anxiety disorders are potential risk factors for having difficulty coping with stressful situations [29]. At the same time, less efficient coping mechanisms can result in the intensification of symptoms when faced with threatening situations. The frequency of specific concerns related to the consequences of the pandemic was the same in those with and without mental illness diagnoses. However, specific threats were identified with different frequencies in all three tested groups. The self-assessed well-being results turned out to be in line with previous results. The highest level of well-being was experienced by individuals with no diagnosis and the lowest by individuals with depression. The low subjective well-being of individuals with depression is not only related to their low well-being, but low well-being at a given point in time can also cause depression in the future [12]. Participants with depression declared significantly lower social well-being compared to healthy ones. In Keye’s definition, social well-being is composed of several different components: social integration, social input, social coherence, social actualization, and acceptance. Social well-being can be understood as a sense of belonging and support from society [2,30].

The number of flourishing individuals within the tested groups was almost identical, which confirms that the assessment of one’s mental health does not depend exclusively on the presence of psychopathology [10]. However, in comparison with individuals with no mental health diagnosis, those with diagnosed mental disorders qualified twice as frequently as languishing.

In previous research, the COVID-19 pandemic was analyzed as a potential risk factor of PTSD with other social factors taken into account. The research performed by Blekas et al. [25] on the healthcare professionals population showed that, on average, 16.7% of participants declared experiencing PTSD symptoms. Tang et al. [31] demonstrated that 2.7% to 9% of isolated students experienced PTSD symptoms. Besides, many studies focusing on psychological consequences of the pandemic are based on questionnaires that measure declarative levels of depression. The attempt to verify the dissemination of depressive disorders and PTSD symptoms based on this type of methodology in cross-sectional studies limits the inference of the impact of the COVID-19 pandemic on mental health, because the baseline indicators of mental disorders dissemination in the general public are also high, often oscillating up to several percent. Ultimately, it is unknown if the obtained results are a consequence of the pandemic or a traditional study on society’s mental state [32].

The analysis of the severity of PTSD indicates that all components of the symptoms were experienced most intensely by individuals with anxiety disorders and least intensely by those with diagnosed depression. It should be made clear that the participants were diagnosed with depression before the outbreak of the pandemic; thus, their illness cannot be considered as a reaction to it. It is quite likely that there are some links between anxiety and severe stress, which is a main component of PTSD, in the difficult situation of the pandemic. Although fear can also be considered to be an accompanying symptom of depression, it might either be specific to the pandemic, or it might not pertain to any situation. One symptom of depression is apathy and lower interest in the outside world, which could explain the low emotional involvement. At the same time, people with depression had the lowest self-assessed well-being.

It is worth noting that even though people with depression declared lower intensification of PTSD symptoms, the obtained results suggest that the severity of those symptoms is objectively high. The results are analogical to the participants of the study without the diagnosis of mental illness, who, just like participants with depression, experienced the psychological effects of the pandemic comparably. This result is incompatible with the hypothesis that the participants diagnosed with depression would experience more stress related to the pandemic in comparison with the healthy people. Even though individuals with depression declared the highest number of concerns, perhaps the additional stressor did not significantly increase the previously experienced stress. We consider several explanations: possibly, it is due to avoiding the stress-coping strategies, including suicidal ideation or suppression as defense mechanisms [33]. Moreover, it is possible that as a result of the lockdown, the participants diagnosed with depression did not have to face everyday life challenges as much as prior to isolation, which could have reduced their stress level. Depression itself as an illness can exceed the coping resources, and every other stressful situation can result in avoiding strategies. The effect of this study can lead us to an important conclusion: psychological support must be provided not only to those in potential high-risk groups, but also to those who were never psychiatrically treated, because the state of their mental health can deteriorate due to the COVID-19 pandemic.

The severity of PTSD symptoms was connected to overall well-being in each group: the lower the level of well-being, the more severe the PTSD symptoms. The most significant component was emotional well-being, which reflects the hedonic traditions [34]. Emotional well-being is not only the result of pleasant and unpleasant situations, but most importantly, it is the result of a cognitive assessment of overall satisfaction in life [35]. Positive re-evaluation of life events is a protective factor against severe stress in difficult situations, regardless of a diagnosis of mental illness.

### 4.1. Limitations

Some limitations of this research must be considered. First of all, the study groups consisted of a small number of participants, which resulted from including in the final analysis only those meeting the criteria of a single diagnosis of either depression or anxiety disorder. The results were obtained from an online survey; thus, the data were not collected under the researchers’ supervision. The majority of participants were women. It has been shown that women are more eager to participate in scientific studies, especially those performed online [36]. Women are also known to be more inclined to share information on their mental health. In addition, they are more willing to reveal their inner emotional processes to others, which can translate into a bigger commitment and desire to participate in the study [37]. Taking into consideration the fact that women are more often diagnosed with PTSD, the conclusions drawn in this study are more specific to women, and one should be careful when interpreting these results in the context of men [38]. Another limitation of this study was the declarative method of gathering information on the diagnosis of mental illness. There is a risk that some participants could have provided us with false information regarding their mental health status. It is possible that some of the participants who declared to have a mental illness had never had it confirmed by a psychiatrist. Similarly, an undetermined number of those who declared not to suffer from any mental illnesses could, in fact, have hidden the information about their diagnosis. The usage of the declarative method can influence the results in a negative way. The most accurate method would be to verify the diagnosis of participants by independent psychiatrists; however, due to the COVID-19 restrictions, this was not possible. At the same time, the comfortable and safe setting of participants’ own homes could have been helpful in reducing the anxiety that comes with revealing information regarding one’s mental health.

### 4.2. Future Directions

The following studies should involve a higher number of study groups with an even distribution of gender. They could also include participants with different mental disorders, such as schizophrenia or bipolar disorder. It would be interesting to compare the severity of depression and its relation to PTSD within the participants before and during the pandemic, because this would be the only way to observe the real impact of the pandemic on participants’ mental health. However, it would require the authors to be in possession of the data collected prior to the pandemic, and the same participants would have to be encouraged to participate in this study again during the pandemic. Within the context of PTSD research, it would be worthwhile to include possible post-traumatic growth.

## 5. Conclusions

People with depression declared the lowest levels of both well-being and the lowest intensity of PTSD symptoms. The lower the level of emotional well-being, the more often PTSD symptoms were present in individuals with no mental health diagnosis, individuals with depression, and individuals with anxiety disorders.

## Figures and Tables

**Table 1:** Sociodemographic characteristic of participants (*n* = 70 in each group).

	Individuals with No Diagnosis *n* (%)	Individuals with Depressive Disorders *n* (%)	Individuals with Anxiety Disorders *n* (%)
**Sex**			
Women	64 (92%)	63 (90%)	69 (98%)
Men	6 (8%)	7 (10%)	1 (2%)
Educational Background			
Elementary Education	0 (0%)	0 (0%)	0 (0%)
Lower Secondary Education	2 (2.9%)	0 (0%)	1 (1.4%)
Vocational Education	2 (2.9%)	4 (5.7%)	2 (2.9%)
Middle School Education	18 (25.7%)	27 (38.6%)	28 (40%)
Higher Education	45 (64.3%)	39 (55.7%)	39 (55.7%)
PhD	3 (4.2%)	0 (0%)	0 (0%)
Employment			
Current employment contract	46 (66%)	34 (48.5%)	30 (42.8%)
Part-time job under contract for specified service	5 (7%)	5 (7.2%)	2 (2.9%)
Full-time job under contract for specified service	2 (2.9%)	9 (12.9%)	3 (4.3%)
Self-employed (sole employee of the company)	3 (4.3%)	7 (10%)	8 (11.4%)
Company owner, in charge of hiring employees	2 (2.9%)	1 (1.4%)	3 (4.3%)
Student	4 (5.7%)	4 (5.7%)	6 (8.6%)
Retired	0 (0%)	0 (0%)	3 (4.3%)
Unemployed	8 (3.2%)	10 (14.3%)	15 (21.4%)
Have you been tested for COVID-19?			
Yes	0 (0%)	1 (1.4%)	0 (0%)
No	70 (100%)	69 (98.6%)	70 (100%)
I am currently undergoing testing	0 (0%)	0 (0%)	0 (0%)
Result of test for COVID-19?			
Positive	0 (0%)	0 (0%)	0 (0%)
Negative	0 (0%)	1 (1.4%)	0 (0%)
Does not apply	70 (100%)	69 (99.6%)	70 (100%)

**Table 2:** Concerns in each group.

Concerns	Individuals with No Diagnosis	Individuals with Depression	Individuals with Anxiety
Possibility of getting infected with coronavirus	35 (50%)	29 (41.4%)	38 (51.4%)
Own death	27 (38.6%)	22 (31.4%)	39 (55.7%)
Relative testing positive for COVID-19	41 (58.6%)	38 (54.3%)	33 (47.1%)
Death of somebody close	56 (80%)	57 (81.4%)	56 (80%)
Tightening of isolation restrictions	21 (30%)	23 (32.9%)	13 (18.6%)
The obligation to undergo quarantine	9 (12.9%)	14 (20%)	2 (2.9%)
The insufficiency of public health care	39 (55.7%)	41 (58.6%)	41 (58.6%)
Unemployment	36 (51.4%)	29 (41.4%)	29 (41.4%)
Significant deterioration of socio-economic situation	38 (54.3%)	39 (55.7%)	28 (40%)
Social isolation/loneliness	21 (30%)	36 (51.4%)	22 (31.4%)
Worsening of family relations	7 (10%)	16 (22.9%)	9 (12.9%)
Home schooling	4 (5.7%)	6 (8.6%)	6 (8.6%)
Too many obligations when returning to normal life	11 (15.7%)	14 (20%)	11 (15.7%)
Average number of concerns	4.92	5.2	4.67

**Table 3:** Analysis of variance in intergroup scheme of well-being between healthy participants, participants with depression, and participants with anxiety disorders (*n* = 70 in each group).

Well-Being	No Diagnosis (1)			Depressive Disorders (2)			Anxiety Disorders (3)			*F*(2, 209)	*η^2^*	Post Hoc
	*M*	*SD*	Min/Max	*M*	*SD*	Min/Max	*M*	*SD*	Min/Max			
Emotional	10.88	4.25	3/18	9.65	3.99	3/18	10.01	4.47	3/18	1.55	0.01	-
Social	15.40	5.69	5/29	11.94	5.56	5/29	13.88	6.50	5/29	5.96 **	0.05	1 > 2
Psychological	22.47	7.73	6/36	19.98	7.82	6/35	21.81	8.18	7/36	1.85	0.01	-
Overall result	48.75	15.75	14/80	41.58	15.02	15/82	45.71	16.58	19/83	3.63 *	0.04	1 > 2

Bonferroni post hoc test: *** *p* < 0.001; ** *p* < 0.01; * *p* < 0.05.

**Table 4:** Participants who were flourishing, languishing, and with average mental health in the three groups.

	Flourishing	Languishing	Average Mental Health
Individuals without mental disorders	10 people—14%	10 people—14%	50 people—72%
Individuals with depressive disorders	11 people—16%	23 people—33%	36 people—51%
Individuals with anxiety disorders	10 people—14%	18 people—26%	28 people—60%

**Table 5:** One-way analysis of variance in the intergroup scheme of PTSD symptoms between healthy participants, participants with depression, and participants with anxiety disorders. (*n* = 70 in each group).

PTSD	No Diagnosis (1)			Depressive disorders (2)			Anxiety Disorders (3)			*F*(2, 209)	*η^2^*	Post Hoc
	*M*	*SD*	Min/Max	*M*	*SD*	Min/Max	*M*	*SD*	Min/Max			
Intrusion	1.64	0.92	0/3.63	1.52	0.98	0/4	2.05	0.98	0/3.75	5.75 **	0.05	2 < 1 < 3
Hyperarousal	1.70	0.94	0/4	1.70	0.89	0/3.86	2.16	0.90	0/3.43	5.84 **	0.05	1 < 2 < 3
Avoidance	1.74	0.89	0/4	1.70	0.81	0/3.83	1.85	0.82	0/3.29	0.58	0.001	-
Overall result	1.69	0.83	0/3.73	1.63	0.81	0/3.73	2.02	0.82	0/3.27	3.01 **	0.04	2 < 3

Bonferroni post hoc test: *** *p* < 0.001; ** *p* < 0.01; * *p* < 0.05.

**Table 6:** Pearson correlation coefficients between well-being and PTSD symptoms (*n* = 70 in each group).

Individuals with No Diagnosis	Intrusion	Hyperarousal	Avoidance	PTSD Overall Result
Emotional well-being	−0.426 ***	−0.427 **	−0.296 *	−0.415 ***
Psychological well-being	−0.180	−0.232	−0.160	−0.209
Social well-being	−0.131	−0.133	−0.063	−0.122
Well-being (overall result)	−0.251 *	−0.278 *	−0.174	−0.259 *
Individuals with depressive disorders				
Emotional well-being	−0.322 ***	−0.495 **	−0.229	−0.384 ***
Psychological well-being	−0.143	−0.334 **	−0.177	−0.234
Social well-being	−0.074	−0.165	−0.138	−0.133
Well-being (overall result)	−0.187	−0.367 ***	−0.204	−0.273 *
Individuals with anxiety disorders				
Emotional well-being	−0.339 ***	−0.334 **	−0.336 **	−0.373 ***
Psychological well-being	−0.154	−0.173	−0.137	−0.172
Social well-being	−0.285 *	−0.264 *	−0.092	−0.246 *
Well-being (overall result)	−0.280 *	−0.279 *	−0.194	−0.282 *

*** correlation significance *p* < 0.001; ** correlation significance *p* < 0.01; * correlation significance *p* < 0.05.

**Table 7:** PTSD predictors (individuals with no diagnosis) (*n* = 70 in each group).

	*Beta*	B	Error B	t	*p*
Well-being (overall result)	−0.259	−0.014	0.006	−2.210	0.031
Constant value		2.370	0.320	7.418	0.000

*R* = 0.259; *R^2^* = 0.067.

**Table 8:** PTSD predictors (individuals with no diagnosis) (*n* = 70 in each group).

	*Beta*	B	Error B	t	*p*	Variance Inflation Factor
Emotional well-being	−0.615	−0.121	0.032	−3.801	0.000	2.202
Psychological well-being	0.030	0.003	0.018	0.180	0.858	2.277
Social well-being	0.269	0.040	0.024	1.673	0.099	2.173
Constant value		2.335	0.298	7.846	0.000	

*R* = 0.465; *R*^2^ = 0.216.

**Table 9:** PTSD predictors (individuals with depression) (*n* = 70 in each group).

	*Beta*	B	Error B	T	*p*
Well-being (overall result)	−0.015	−0.237	0.006	−2.344	0.022
Constant value		2.259	0.281	8.037	0.000

*R* = 0.273; *R*^2^ = 0.075.

**Table 10:** PTSD predictors (individuals with depression) (*n* = 70 in each group).

	*Beta*	B	Error B	t	*p*	Variance Inflation Factor
Emotional well-being	−0.450	−0.092	0.033	−2.788	0.007	2.032
Psychological well-being	0.032	0.003	0.018	0.190	0.850	2.241
Social well-being	0.082	0.012	0.021	0.580	0.564	1.575
Constant value		2.317	0.274	8.463	0.000	

*R* = 0.393; *R*^2^ = 0.154.

**Table 11:** PTSD predictors (individuals with anxiety) (*n* = 70 in each group).

	*Beta*	B	Error B	T	*p*
Well-being (overall result)	−0.282	−0.014	0.006	−2.424	0.018
Constant value		2.663	0.280	9.504	0.000

*R* = 0.282; *R*^2^ = 0.080.

**Table 12:** PTSD predictors (individuals with anxiety) (*n* = 70 in each group).

	*Beta*	B	Error B	t	*p*	Variance Inflation Factor
Emotional well-being	−0.406	−0.075	0.028	−2.693	0.009	1.775
Psychological well-being	0.163	0.016	0.016	1.005	0.319	1.789
Social well-being	−0.125	−0.016	0.019	−0.825	0.412	2.058
Constant value		2.634	0.276	9.555	0.000	

*R* = 0.393; *R*^2^ = 0.154.

## Data Availability

The data that support the findings of this study are available on request from the corresponding author [PG].
